# Dietary Qi-Weng-Huangbo powder enhances growth performance, diarrhoea and immune function of weaned piglets by modulating gut health and microbial profiles

**DOI:** 10.3389/fimmu.2023.1342852

**Published:** 2023-12-19

**Authors:** Chuanpi Xiao, Kai Li, Chunran Teng, Zeou Wei, Jiaheng Li, Shunfeng Zhang, Lei Liu, Huiyuan Lv, Ruqing Zhong

**Affiliations:** ^1^State Key Laboratory of Animal Nutrition and Feeding, Institute of Animal Sciences, Chinese Academy of Agricultural Sciences, Beijing, China; ^2^Precision Livestock and Nutrition Unit, Gembloux Agro-Bio Tech, University of Liège, Gembloux, Belgium; ^3^School of Agriculture and Food Science, University College Dublin, Dublin, Ireland; ^4^Peking Centre Technology Co., LTD, Beijing, China

**Keywords:** diarrhoea, gut health, herbal medicine, immune function, weaned piglets

## Abstract

**Introduction:**

The evolution of nutritional strategies to improve the gut health and microbiota profiles of early-weaned piglets is essential to reduce diarrhoea caused by weaning stress. Therefore, the aim of this study was to determine the effects of dietary supplementation of Qi-Weng-Huangbo powder, a traditional herbal medicine consisting of a mixture of *Pulsatilla chinensis*, *Chinese Schneid* and *Astragalus* extracts (PCE), on the growth performance, diarrhoea rate, immune function and intestinal health of weaned piglets.

**Methods:**

162 piglets were randomly assigned to the CON group (no PCE added), the PCEL group (300 mg/kg PCE) and the PCEH group (500 mg/kg PCE) at the end of the third week post farrowing. There were 9 replicates of each group with 6 pigs per replicate. The experiment lasted for 28 days and sampling was performed on the final day.

**Results:**

The results showed that the PCE diet increased the average daily gain (ADG) and final body weight (BW) compared to the CON group. Both supplemented doses of PCE reduced the faecal scores of piglets, and the diarrhoea rate in the PCEL group was significantly lower than that in the CON group. The application of PCE diets promoted the development of the spleen in piglets and up-regulated serum immunoglobulin concentrations to enhance immune function, which was also reflected in the down-regulated gene expression of the colonic TLR/MyD88/NF-κB pathway. Supplementation with PCE improved intestinal morphology, and all doses of PCE significantly increased villus height (VH) in the ileum, whereas colonic crypt depth (CD) was significantly lower in the PCEH group than in the CON group. The PCEH diet significantly increased the levels of valeric and isovaleric acid in the colon content. Dietary PCEH also improved the colonic microbial community profile, reflected by a significant increase in Shannon’s index compared with CON group. The abundance of *Veillonellaceae* and *Rhodospirillales* was significantly increased in the PCEH group at the family level.

**Discussion:**

In conclusion, dietary PCE reduced diarrhoea rates, improved growth performance and enhanced immune function in weaned piglets. These improvements were potentially supported by altered ileum and colonic morphology, elevated colonic VFA levels, and modulation of colonic microbial profiles.

## Introduction

Early weaning of piglets, typically occurring at 3 to 4 weeks of life, is a widely employed strategy aimed at maximizing economic gains through the efficient repopulation of sows (1). However, this practice induces weaning stress as a consequence of separation from the sow and dietary alterations, thereby instigating disruptions in the microbial composition and metabolic processes within the piglet’s gastrointestinal tract (2, 3). Consequently, the structural and functional integrity of the gut may be compromised, leading to the atrophy of intestinal villi and heightened mucosal permeability (4). These detrimental effects ultimately result in a diminished immune response and the manifestation of diarrheal symptoms in piglets. In the global context of the ban on antibiotics in feed, concerns regarding bioresistance have elevated the significance of these threats (5). Thus, it is imperative to employ nutritional strategies to enhance piglet gut health during the early weaning period.

Qi-weng-Huangbo powder, the name comes from the Chinese pronunciation of the abbreviated names of three Chinese herbs, comprising a mixture of *Pulsatilla chinensis*, *Chinese Schneid*, and *Astragalus* extract (PCE), serves as a traditional Chinese herbal compound (6). *Pulsatilla chinensis*, a perennial herb belonging to the *Pulsatilla* genus in the buttercup family, is utilized in traditional Chinese medicine for its efficacy in treating bacterial infections (7). The root of this herb contains *Pulsatilla chinensis* saponin, which serves as its primary active ingredient. Additionally, *Chinese Schneid* and *Astragalus* are sourced from the bark and root of their respective plants. The active component of *Chinese Schneid* is berberine hydrochloride, whereas *Astragalus* contains astragalus polysaccharide as its active ingredient (8, 9). Recent pharmacological research has elucidated the modulatory effects of saponins derived from *Pulsatilla chinensis* on various biological processes, including apoptosis, immunomodulation, neuroprotection, and anticancer mechanisms (10). Previous investigations have demonstrated that the administration of *Pulsatilla chinensis* extract can effectively restore the integrity of the colonic mucosal barrier in rats by preserving the homeostasis and diversity of the intestinal microbiota (11). Furthermore, the therapeutic potential of *Pulsatilla chinensis* extract in the treatment of colitis has been attributed to its ability to enhance the levels of colonic volatile fatty acids (12). Berberine hydrochloride exhibits anti-inflammatory and immunosuppressive properties, while astragalus polysaccharide has been found to enhance animal growth performance by mitigating oxidative stress (13).

In recent years, there has been increasing utilization of single or combined herbs as feed additives, whereby the immune function of weaned piglets has been enhanced through the administration of astragalus and ginseng polysaccharides for instance (14). Egg quality in laying hens has also been improved by employing cinnamon and rosemary essential oils, while antioxidant activity in ducks has been elevated through the utilization of *Pulsatilla chinensis* and *Eucommia* extracts (15, 16). Additionally, a number of studies have been conducted to investigate the potential of *Pulsatilla chinensis* extracts in ameliorating intestinal inflammation and mitigating LPS challenge-induced intestinal damage in piglets. Nevertheless, there remains a lack of comprehensive research examining the impact of traditional herbal formulations on growth performance, diarrhoea rate, and intestinal health in weaned piglets (17). As such, the objective of this study was to assess the effects of applying PCE on the immune function, intestinal development, and colonic microbial profiles in weaned piglets fed varying dosages.

## Materials and methods

The Animal Ethics Committee of the Institute of Animal Sciences, Chinese Academy of Agricultural Sciences approved the experimental protocol (Ethics Approval Code: IAS2022-155).

### Experimental design and treatments

The Qi-Weng-Huangbo powder, sourced from Peaking Centre Technology Co., Ltd, consisted of *Pulsatilla chinensis*, *Chinese Schneid*, and *Astragalus* in the ratio 4:3:3 respectively. The preparation process involved boiling the mixture for three hours to obtain a solution, followed by filtration and subsequent drying of the filtrate. Starch was then added to the resulting product, at a weight equivalent to 50% of the original herb, and thoroughly mixed to yield the Qi-Weng-Huangbo powder.

A total of 162 Yorkshire x (Duroc x Landrace) piglets, with similar body weights, were randomly allocated into three groups consisting of nine replicates of six pigs each, with an equal distribution of females and males, weaning in the last day of the third week. The control group (CON) was fed a basal diet and the low-dose group (PCEL) received a basal diet supplemented with 300 mg/kg of PCE, while the high-dose group (PCEH) received a basal diet supplemented with 500 mg/kg of PCE.

The animal experiment was conducted at the Dabeinong Research Centre Farm in Tangshan, China, with unrestricted access to water and feed. The basal diet used in the experiment (as outlined in **Table 1**) adhered to the nutritional requirements recommended by the National Research Council. The animals were housed in an environment maintained at a temperature of 30°C and a relative humidity of 70%, with a daily light period of 12 hours. To ensure the validity of the data, the use of antibiotics was strictly prohibited throughout the duration of the experiment. The experiment spanned a total of 28 days.

**Table 1:** Composition and nutritional levels of the basal diet.

Ingredient	Contents, %
Corn	52.55
Soybean meal	18.00
Whey powder	5.00
Extruded soybean	6.50
Flour	10.00
NaCl	0.12
Fish meal	3.50
Limestone	1.20
Dicalcium phosphate	0.75
Soybean oil	1.20
Vitamin premix	0.20
Mineral premix	0.20
DL-Methionine	0.23
L-Lysine (98%)	0.58
Total	100.00
Nutrient level	
Digestible Energy, MJ/kg	14.51 MJ/kg
Crude protein, %	18.99
Lysine, %	1.30
Methionine, %	0.51
Calcium, %	0.84
Total phosphorus, %	0.62
Available phosphorus, %	0.42

Provided per kilogram of compound diet: vitamin A, 12000 IU; vitamin D3, 600 IU; vitamin E, 20 mg; VK, 3.2 mg; vitamin B1, 3.2 mg; vitamin B2, 3.6 mg; nicotinic acid, 50mg; pantothenic acid, 20mg; vitamin B6, 4.3 mg; biotin, 0.22 mg; folic acid, 2.2 mg; vitamin B12, 0.017 mg; I, 1.25 mg; Fe, 120 mg; Mn, 80 mg; Se, 0.3 mg; Zn, 110 mg. Nutrition level was calculated value.

### Growth performance

The study collected and recorded body weight data and feed intake per treatment using an automated system. The body weights of all piglets were measured on the first day and 28th day of the trial to determine the initial body weight (Inital BW), final body weight (Final BW), and daily feed intake (ADFI). The feed conversion ratio (FCR) was calculated as ADFI/Final BW - Inital BW.

### Faecal scores and diarrhoea rates

Faecal scoring was conducted to assess shape and water content, with scores ranging from 0 to 5. Diarrhoea was identified when the faecal score exceeded 2. The scoring scale employed in this study encompassed the following categories: a score of 0 denoted normal solid faeces, a score of 1 indicated somewhat moist faeces that still maintained a solid shape, a score of 2 signified faeces that were beginning to lose their solid consistency, a score of 3 represented the onset of diarrhoea characterized by the absence of solid form, a score of 4 indicated a more severe form of diarrhoea where the faeces exhibited a runny consistency, and finally, a score of 5 denoted faeces that were completely watery, representing the most severe manifestation of diarrhoea. The total number of diarrhoeic piglets was recorded daily throughout the experimental period. The diarrhoea rate was calculated as: number of diarrhoeic pigs/(total number of pigs × number of days) × 100%.

### Sample collection

On day 28, sample collection was conducted, whereby one male piglet was randomly chosen from each replicate. The piglets were weighed, and blood samples were extracted from the jugular vein. The serum was obtained through low-temperature centrifugation (9,000 × *g*) for 10 minutes and subsequently stored in a freezer at -20°C. Ileum and colon samples measuring 1 cm in length were sectioned at identical locations, gently rinsed with PBS, and promptly submerged in a 10% formalin solution, prior to dehydration. The colonic mucosa and colonic content were placed into separate sterile tubes and rapidly frozen in liquid nitrogen and stored in a -80°C freezer for subsequent index analysis. The heart, liver, spleen, lungs, and kidneys were individually weighed and used to calculate the organ index, which is determined by dividing the organ weight by the weight of the pig and multiplying it by 100%.

### Determination of immunoglobulin

The levels of immunoglobulin A (IgA), immunoglobulin G (IgG), and immunoglobulin M (IgM) in the serum were assessed using Enzyme-Linked Immunosorbent Assay (ELISA) kits provided by MLBIO (Shanghai, China). All experimental procedures were conducted in strict accordance with the manufacturer’s instructions. The data exhibited an intra-batch coefficient of variation (CV) of less than 5% and an inter-batch CV of less than 8%.

### Morphology of the ileum and colon

The intestinal samples were immersed in a 10% formalin solution for a duration of 24 hours to ensure fixation. Subsequently, the intestinal segments underwent dehydration and were embedded in paraffin wax. Tissue slices measuring 5 µm in thickness were then prepared using a microtome and subjected to staining with haematoxylin and eosin. The resulting sections were examined and captured using a Nikon 80i microscope, and the software Image J was employed to analyse the intestinal morphology. Villus height (VH) was determined as the measurement from the apex of the villus to the junction of the adjacent crypt, while crypt depth (CD) was defined as the extent of the depression between neighbouring villi. Ten representative data points from each sample were selected for statistical analysis.

### Analysis of volatile fatty acids

The analysis of VFAs involved loading approximately 1 g of colonic content into a 10 mL centrifuge tube, thoroughly mixing it with 5 mL of distilled water, and then centrifuging it at 9,000 × *g* for 10 min after 1 h at 4°C. Following this, 900 μL of the supernatant was combined with 100 μL of 25% metaphosphoric acid and allowed to stand for 2 h at 0°C. After another round of centrifugation for 10 min, the resulting supernatant was subjected to gas chromatography (Agilent 7890) for the analysis.

### RNA isolation and real-time quantitative PCR

Total colon RNA was extracted using Trizol reagent (Invitrogen, San Diego, USA), and the concentration and purity of each RNA sample were assessed using a NanoDrop spectrophotometer (ND-2000, Thermo Scientific, Wilmington, USA). The integrity of the RNA was evaluated by 1% agarose gel electrophoresis. Reverse transcription was performed on 1 μg of total RNA using the PrimeScript^®^ RT kit (RR047A, TaKaRa, Japan). Subsequently, gene expression was determined by analysing 1 μg of total RNA with TB Green Premix Ex Taq (RR820A, Takara, Japan) on an ABI 7500 real-time PCR system (Thermo Scientific, Wilmington, USA) for RT-PCR analysis. The reaction program consisted of pre-denaturation at 95°C for 10 s, followed by denaturation at 95°C for 5 s, and annealing and extension at 60°C for 40 s. Each reaction was replicated in three wells, and the primer sequences are presented in **Table 2**. The amplification efficiency of the primers was determined by employing a standard curve. The specificity of the amplification products was confirmed through melting curves. The expression of target genes was standardised using the geometric mean of glyceraldehyde-3-phosphate dehydrogenase (*GAPDH*) expression. The relative expression levels of each target gene were assessed using the 2^-ΔΔct^ method.

**Table 2:** Primer sequences used for real-time quantitative PCR.

Gene	Primer sequence, 5′→ 3′	Size (bp)
*IL-4*	F: CAGCGAGAAAGAACTCGTG	117
	R: TGTAGATGTGCCGAAGCA	
*IL-6*	F: TCCAATCTGGGTTCAATCA	174
	R: TCTTTCCCTTTTGCCTCA	
*IL-8*	F: TACGCATTCCACACCTTTC	100
	R: GGCAGACCTCTTTTCCATT	
*TLR2*	F: GCCATGTGCTTTGTGGA	58
	R: TTCCTTGGAGAGGCTGATT	
*TLR5*	F: CCGAACCAGCAACCTTT	135
	R: GTGGCATTTCCTTCTGTGA	
*TLR9*	F: TGCTTCCACCTGTGCCT	137
	R: ACCCGCAGCTCGTTGTA	
*NF-κB p65*	F: AGGAGCGGAAGTCAGGA	139
	R: GCTATGTGCAGGGGTGTAG	
*MYD88*	F: CGTCGGATGGTAGTGGTTG	100
	R: TCTGATGGGCACCTGGA	
*GADPH*	F: ACTCACTCTTCCACTTTTGATGCT	100
	R: TGTTGCTGTAGCCAAATTCA	

IL-4, interleukin 4; IL-6, interleukin 6; IL-8, interleukin 8; TLR2, toll-like receptor 2; TLR5, toll-like receptor 5; TLR9, toll-like receptor 9; NF-κB p65, nuclear factor kappa-B p65; MYD88, adaptor protein myeloid differentiation primary response 88; GADPH, glyceraldehyde-3-phosphate dehydrogenase.

### 16S sequencing of colonic microorganisms

Total genomic DNA from the contents of the colon was extracted using the E.Z.N.A. Soil DNA Kit (Omega Bio-Tek, Norcross, CA, USA). Extracted DNA was assessed for both quantity and purity using a NanoDrop spectrophotometer (ND-2000, Thermo Scientific, Wilmington, USA). The integrity of the DNA was determined through 1% agarose gel electrophoresis, followed by PCR amplification of the bacterial 16S rRNA gene using specific primers 338F and 806R (GeneAmp9700 produced by ABI) to target the V3-V4 variable region. The amplification program was described as follows: the reaction at 95°C for 3 min was followed by 27 cycles (95°C for 30 s, 55°C for 30 s of annealing, and 72°C for 30 s), and the final step was an extension at 72°C for 10 min, while the PCR was performed in 20 μL of a mixed system containing 10 ng of template DNA. The resulting PCR products were then recovered on a 2% agarose gel and purified using the AxyPrep DNA Gel Extraction Kit (Axygen Biosciences, Union City, CA, USA) before quantification using the QuantiFluor™-ST assay (Promega, USA).

The purified amplification products were combined in equimolar proportions and underwent paired-end sequencing (2 × 300) on an Illumina MiSeq PE300 platform (Illumina, San Diego, CA, USA). The raw sequences were subjected to quality screening using Quantitative Insights Into Microbial Ecology 2 (QIIME2) software. Subsequently, the demultiplexed sequences of each sample were filtered, trimmed, denoised, and merged. Chimeric sequences were identified and eliminated to generate a table containing characteristics of amplicon sequence variation. Based on the 338F/806R primers, the database obtained in the previous step was trimmed to the V3-V4 region to obtain the species classification table.

### Data analysis

All data were expressed as the mean and total standard error of the mean, and data were counted and analysed in replicates. Data were analysed by one-way ANOVA using the software SPSS 22.0 (SPSS. Inc., Chicago, USA). Statistical differences in the data were considered to be present when *P* < 0.05.

## Results

### Growth performance and organ index

**Table 3** presents the findings regarding the impact of various doses of PCE on the performance of weaned piglets. The results indicated that all doses of PCE had a significant positive effect on ADG (*P* = 0.002) and final BW (*P* = 0.002) of the piglets. Furthermore, the inclusion of PCEL in the diet resulted in a significant increase in ADFI compared to the CON group (*P* = 0.034). Additionally, **Table 4** demonstrated that the administration of extracts in the diet had a significant effect on the organ indexes of the spleen in piglets (*P* = 0.005), while the organ indexes of the heart, liver, lungs, and kidneys did not exhibit significant changes (*P* > 0.05).

**Table 3:** Effects of dietary PCE on the growth performance of the weaned piglets^1,2,3^.

Item	Diet			SEM	*P*-value
	CON	PCEL	PCEH		
Inital BW, kg	7.09	7.14	7.16	0.12	0.522
Final BW, kg	16.50^b^	18.24^a^	17.55^a^	1.02	0.002
ADFI, kg	0.56^b^	0.61^a^	0.58^ab^	0.04	0.034
ADG, kg	0.35^b^	0.41^a^	0.39^a^	0.03	0.002
FCR, kg/kg	1.59	1.50	1.51	0.11	0.257

^1^Means in a row with no common superscript are significantly different (P < 0.05).

^2^Means are based on 6 replicates per treatment (n = 9).

^3^SEM, standard error of the mean; BW, body weight; ADFI, average daily feed intake; ADG, average daily gain; FCR, feed conversion ratio; CON, piglets fed with the basal diet; PCE, Mixture of Pulsatilla chinensis, Chinese Schneid and Astragalus extract; PCEL, piglets fed with 300mg/kg PCE; PCEH, piglets fed with 500mg/kg PCE.

**Table 4:** Effects of dietary PCE on the organ indexes of the weaned piglets^1,2,3^.

Item	Diet			SEM	*P*-value
	CON	PCEL	PCEH		
Heart, %	0.54	0.54	0.51	0.03	0.386
Liver, %	2.81	2.78	2.78	0.06	0.962
Spleen, %	0.21^b^	0.28^a^	0.28^a^	0.04	0.005
Lung, %	1.02	1.07	1.09	0.68	0.320
Kidney, %	0.47	0.49	0.46	0.02	0.334

^1^Means in a row with no common superscript are significantly different (P < 0.05).

^2^Means are based on 6 replicates per treatment (n = 9).

^3^SEM, standard error of the mean; CON, piglets fed with the basal diet; PCE, Mixture of Pulsatilla chinensis, Chinese Schneid and Astragalus extract; PCEL, piglets fed with 300mg/kg PCE; PCEH, piglets fed with 500mg/kg PCE.

### Faecal scores and diarrhoea rates

**Figure 1:**
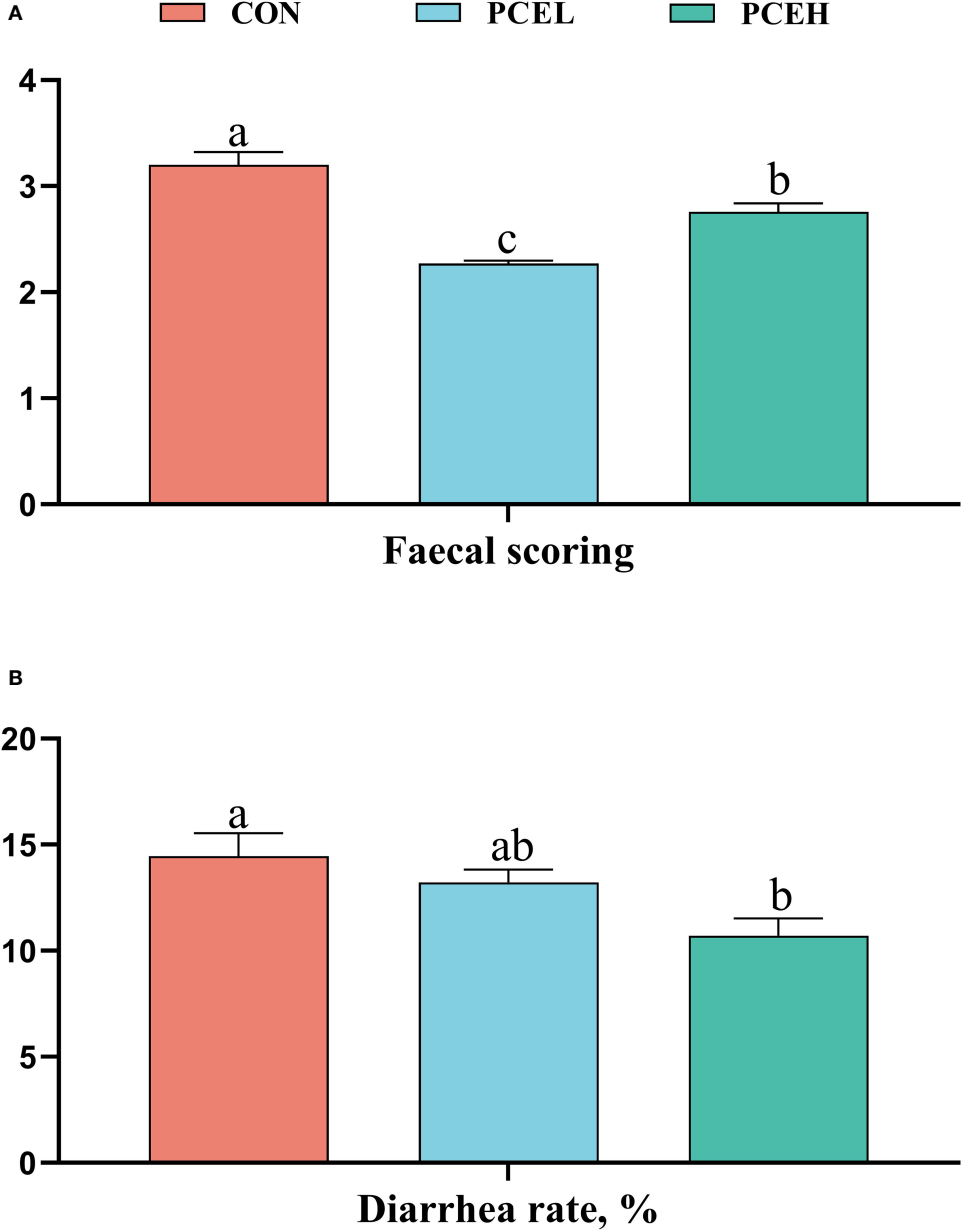
Effect of dietary PCE on faecal scoring **(A)** and diarrhoea rate **(B)** of the weaned piglets. Means with no common superscripts differ significantly (*P* < 0.05). CON, piglets fed with the basal diet; PCE, Mixture of *Pulsatilla chinensis*, *Chinese Schneid* and *Astragalus extract*; PCEL, piglets fed with 300mg/kg PCE; PCEH, piglets fed with 500mg/kg PCE.

**Figure 1** provides evidence supporting the impact of PCE on the faecal scores and diarrhoea rates of piglets. The data clearly demonstrated that PCE significantly decreased the faecal scores of piglets (*P* < 0.001) and exhibited a dose-dependent positive effect. Furthermore, the diarrhoea rate in the PCEL group was significantly lower compared to the CON group (*P* = 0.017).

### Serum immunoglobulins

**Figure 2:**
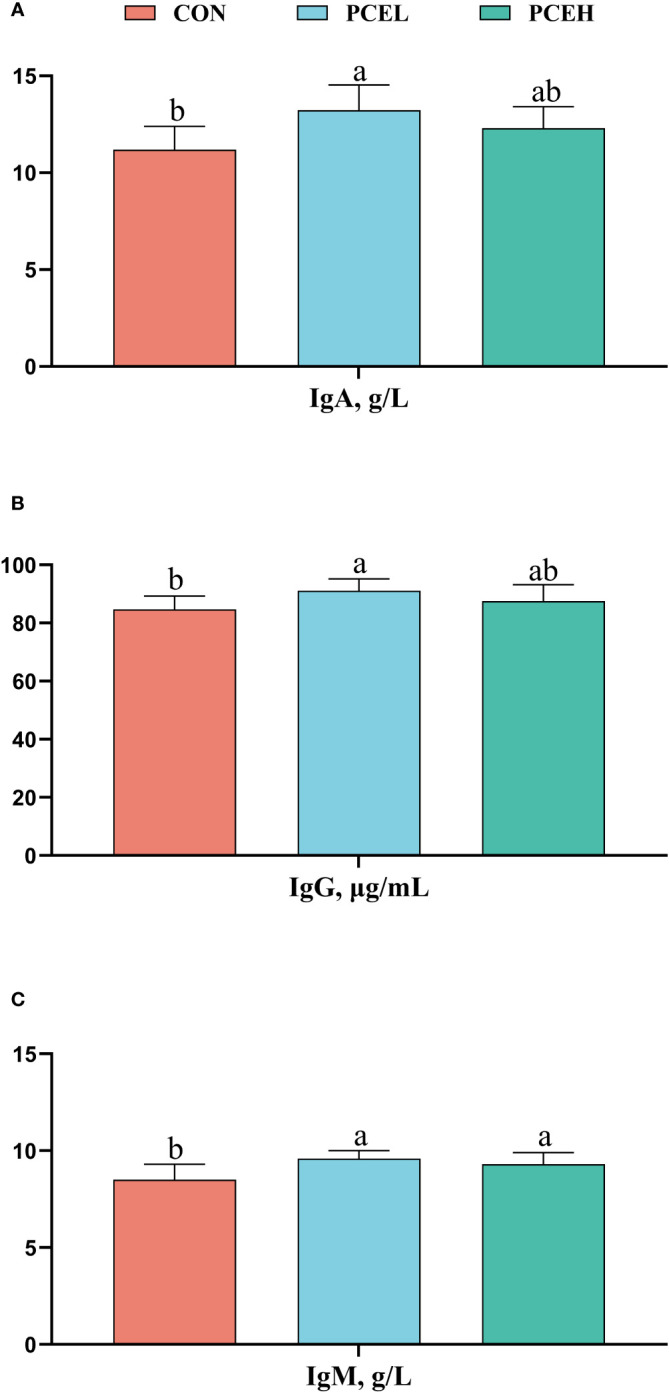
Effects of dietary PCE on serum immunoglobulin A **(A)**, immunoglobulin G **(B)**, and immunoglobulin M **(C)** concentrations of the weaned piglets. Means with no common superscripts differ significantly (*P* < 0.05). Ig, immunoglobulin; CON, piglets fed with the basal diet; PCE, Mixture of *Pulsatilla chinensis*, *Chinese Schneid* and *Astragalus extract*; PCEL, piglets fed with 300mg/kg PCE; PCEH, piglets fed with 500mg/kg PCE.

The influence of PCE on the serum immunoglobulins of weaned piglets is presented in **Figure 2**. The PCEL group exhibited a significant increase in the levels of serum IgA, IgG, and IgM in piglets compared to the CON group (*P* < 0.05). Additionally, the PCEH group demonstrated a significant beneficial effect on IgM (*P* = 0.036).

### Morphometric analysis of the ileum and colon

**Figure 3:**
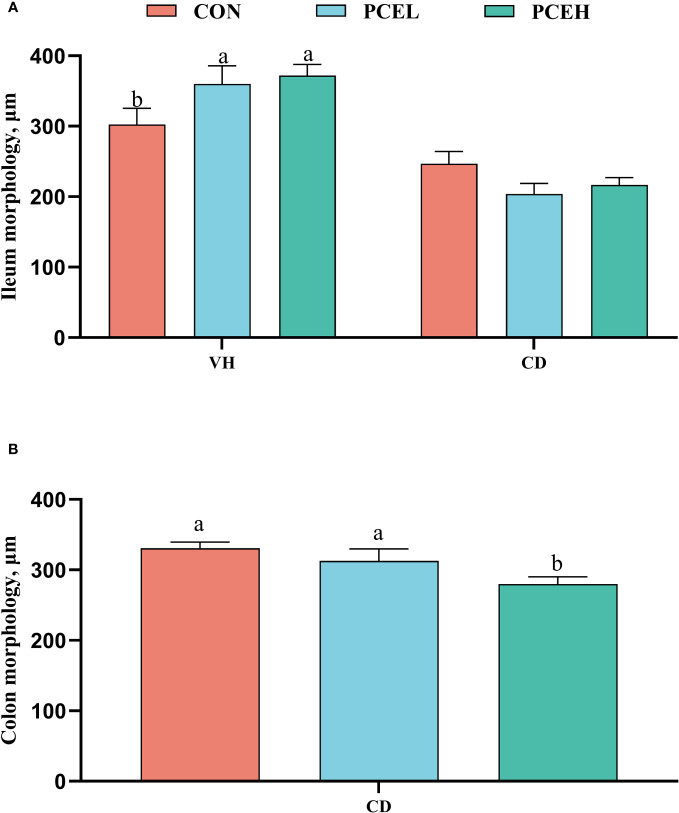
Effects of dietary PCE on morphology of the ileum **(A)** and colon **(B)** of the weaned piglets. Means with no common superscripts differ significantly (*P* < 0.05). VH, villus height; CD, crypt depth; CON, piglets fed with the basal diet; PCE, Mixture of *Pulsatilla chinensis*, *Chinese Schneid* and *Astragalus extract*; PCEL, piglets fed with 300mg/kg PCE; PCEH, piglets fed with 500mg/kg PCE.

The findings presented in **Figure 3** illustrate the impact of diet on the intestinal morphology of weaned piglets. Specifically, the administration of PCE at a dosage of 500 mg/kg resulted in a significant increase in ileal, as well as a decrease in colonic CD, when compared to the normal diet (*P* < 0.05). Furthermore, the PCEL group exhibited significantly higher ileal compared to the CON group (*P* < 0.05).

### Volatile fatty acids concentration of the colonic content

The effect of PCE diets on the concentration of VFAs in the colonic content of weaned piglets is presented in **Table 5**. Concentrations of valeric acid and isovaleric acid were significantly higher (*P* < 0.05) in the PCEH group compared to the CON group. However, the low-dose PCE diet did not show a trend towards significant changes.

**Table 5:** Effects of dietary PCE on colonic concentrations of VFAs of the weaned piglets^1,2,3^.

Item	Diet			SEM	*P*-value
	CON	PCEL	PCEH		
Acetic acid, mg/kg	5443.85	5586.43	4862.18	250.00	0.233
Propionic acid, mg/kg	2501.81	2839.70	2744.73	130.00	0.133
Butyric acid, mg/kg	1583.40	1859.29	1846.11	135.00	0.320
IsoButyric acid, mg/kg	159.49	155.44	166.83	7.60	0.262
Valeric acid, mg/kg	403.04^b^	407.56^b^	508.31^a^	21.50	0.002
IsoValeric acid, mg/kg	182.80^b^	193.64^b^	250.82^a^	10.20	0.023

^1^Means in a row with no common superscript are significantly different (P < 0.05).

^2^Means are based on 6 replicates per treatment (n = 9).

^3^SEM, standard error of the mean; CON, piglets fed with the basal diet; PCE, Mixture of Pulsatilla chinensis, Chinese Schneid and Astragalus extract; PCEL, piglets fed with 300mg/kg PCE; PCEH, piglets fed with 500mg/kg PCE.

### Inflammatory factor-related gene expression

**Figure 4:**
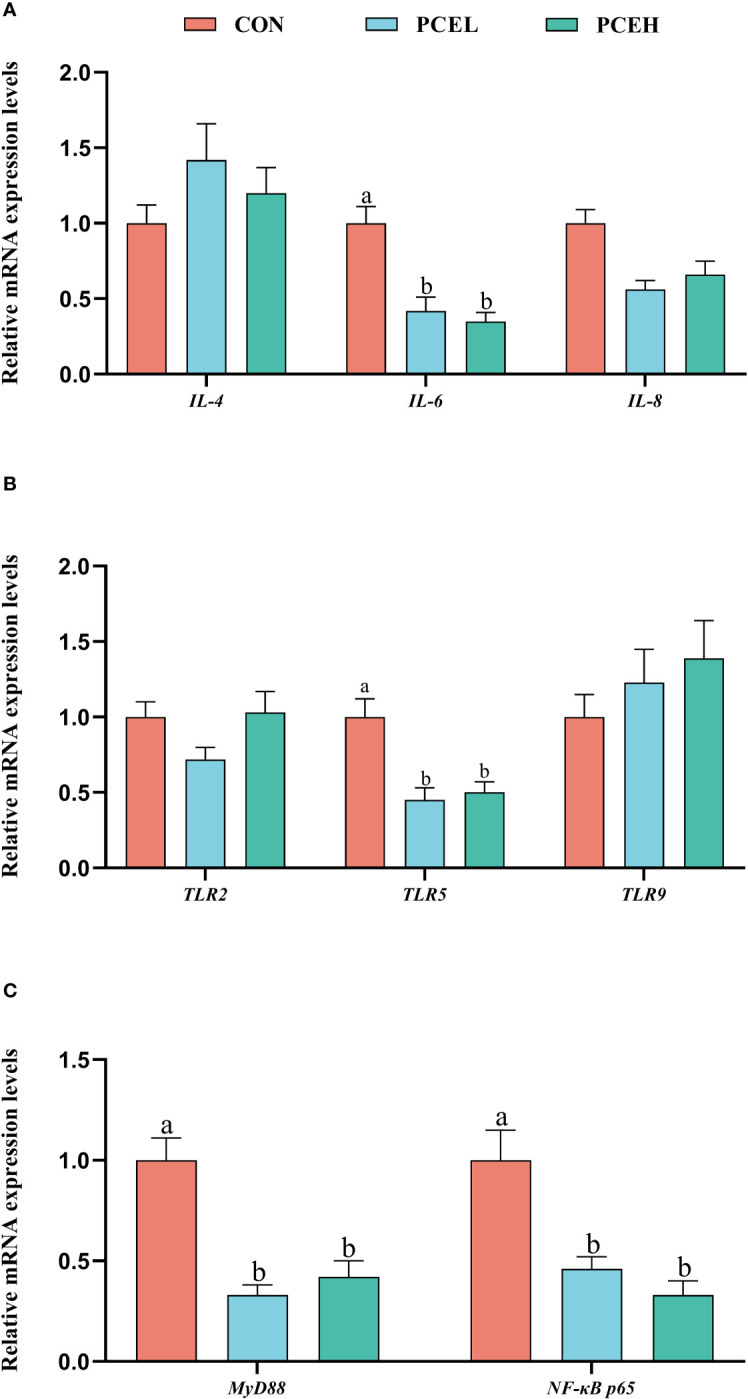
Effects of dietary PCE on the gene expression levels of immune cytokines and TLR/MyD88/NF-κB pathway in the colon of the weaned piglets. Means with no common superscripts differ significantly (*P* < 0.05). *IL,* interleukin; *TLR*, toll-like receptor; *NF-κB p65*, nuclear factor kappa-B p65; *MYD88*, adaptor protein myeloid differentiation primary response 88; CON, piglets fed with the basal diet; PCE, Mixture of *Pulsatilla chinensis*, *Chinese Schneid* and *Astragalus extract*; PCEL, piglets fed with 300mg/kg PCE; PCEH, piglets fed with 500mg/kg PCE.

**Figure 4** demonstrates a significant downregulation of gene expression levels of the pro-inflammatory factor interleukin 6 *(IL-6*) in the colon of piglets in the PCEL and PCEH groups, as compared to CON piglets. Additionally, the gene expression levels of toll-like receptor 5 (*TLR5*), adaptor protein myeloid differentiation primary response 88 (*MyD88*), and nuclear factor kappa-B p65 (*NF-κB p65*) were also significantly downregulated (*P* < 0.05). These findings suggested that the application of PCE lead to the downregulation of gene expression in the TLR/MyD88/NF-κB pathway.

### Colonic microbial profiles

**Figure 5:**
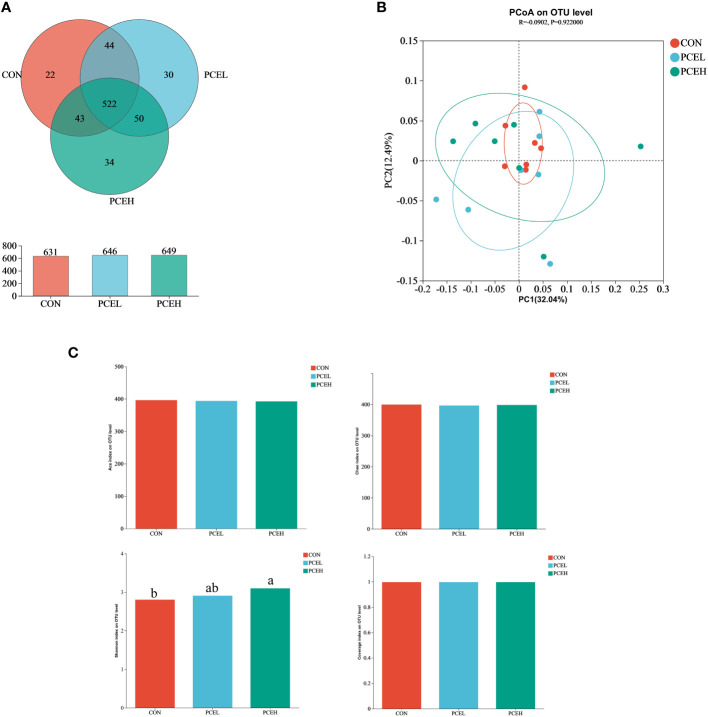
Effects of dietary PCE on colonic microbiota diversity of the weaned piglets. **(A)** Venn diagram based on the number of OTUs per group; **(B)** PCoA based on unweighted Unifrac distance; **(C)** α diversity based on the Ace, Chao1, Shannon and Coverage indices. Means with no common superscripts differ significantly (*P* < 0.05). CON, piglets fed with the basal diet; PCE, Mixture of *Pulsatilla chinensis*, *Chinese Schneid* and *Astragalus extract*; PCEL, piglets fed with 300mg/kg PCE; PCEH, piglets fed with 500mg/kg PCE.

In order to assess the impact of dietary PCE on colonic microorganisms, we conducted high-throughput sequencing of colonic content in weaned piglets using 16s rRNA. Based on the Venn diagram (**Figure 5A**), the CON, PCEL, and PCEH groups exhibited 631, 646, and 649 operational taxonomic units (OTUs) respectively, with 532 OTUs shared among all three colon microbial communities. To analyse beta diversity, we employed unweighted Unifrac distance PCoA analysis to demonstrate that the dietary treatments did not lead to a more distinct separation of colonic microbiota (**Figure 5B**). The alpha-diversity of colonic microorganisms was evaluated using Ace, Chao, Shannon, and Coverage indices (**Figure 5C**), with the Shannon index demonstrating a significant increase in the PCEH group compared to the control group. These findings indicate that the high dose PCE diet has a positive impact on the diversity of colonic microbial populations in piglets.

**Figure 6:**
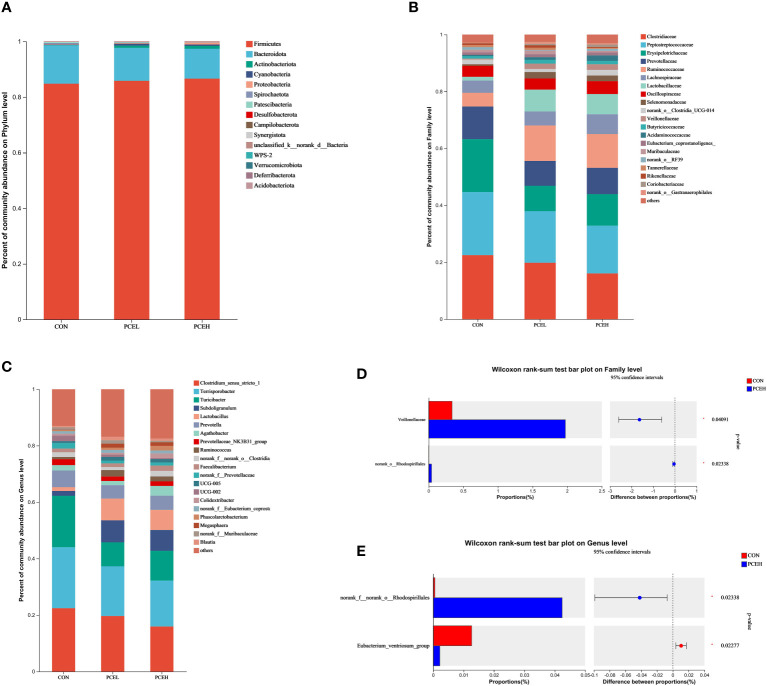
Effects of dietary PCE on colonic microbiota composition of the weaned piglets. **(A)** Microbial composition at the phylum level; **(B)** Microbial composition at the family level; **(C)** Microbial composition at the genus level; **(D)** Relative abundances at the family level; **(E)** Relative abundances at the genus level; * indicates significant difference (*P* < 0.05). CON, piglets fed with the basal diet; PCE, Mixture of *Pulsatilla chinensis*, *Chinese Schneid* and *Astragalus extract*; PCEL, piglets fed with 300mg/kg PCE; PCEH, piglets fed with 500mg/kg PCE.

At the phylum level, statistical analysis revealed that the predominant phyla, *Firmicutes*, *Bacteroidetes*, and *Actinobacteriota* accounted collectively for over 95% of the overall microbial community (**Figure 6A**). At the family level, it is evident that the top five most dominant families, namely *Clostridiaceae*, *Peptostreptococcaceae*, *Erysipelotrichaceae*, *Prevotellaceae*, and *Ruminococcaceaae*, collectively accounted for at least 60% or more of the overall microbial community (**Figure 6B**). When considering microorganisms at the genus level, the primary dominant genera were identified as *Clostridium sensu stricto 1*, *Terrisporobacter*, *Turicibacter*, *Subdoligranulum*, *Prevotella*, and *Lactobacillus* (**Figure 6C**). The results of the Wilcoxon differentiation analysis revealed a statistically significant increase in the abundance of *Veillonellaceae* and *Rhodospirillales* at the family level in the PCEH group compared to the CON group (**Figure 6D**). Furthermore, at the genus level, the abundance of *Rhodospirillales* was significantly higher in the PCEH group compared to the CON group, while the abundance of *Eubacterium ventriosum* was significantly lower in the PCEH group compared to the CON group (**Figure 6E**).

## Discussion

Previous research has demonstrated that the utilization of plant extracts has the potential to enhance animal performance through the modulation of gut health and the improvement of immune function (18). For instance, the administration of *Gynura procumbens*, *Rehmannia glutinosa*, and *Scutellaria baicalensis* has been found to ameliorate diarrhoea in weaned piglets, consequently leading to enhanced growth performance (19). Additionally, the incorporation of *Camellia sinensis* and *Punica granatum* extracts into the drinking water has been shown to effectively counteract *E. coli* infections, thereby preserving the intestinal health of weaned piglets (20). The stress experienced during the weaning process can lead to diarrhoea, compromised immune function, and hindered growth in piglets (21). In line with previous research, the utilization of PCE diets resulted in enhanced ADG and ADFI among weaned piglets, which was directly associated with a reduction in the incidence of diarrhoea. The spleen, an essential immune organ in animals, plays a crucial role in modulating lymphatic and immune-related cytokines to exert anti-inflammatory effects (22). The organ index of the spleen serves as an indicator of the potential immune function of the animal (23). The present study substantiates the efficacy of the PCE extract in enhancing the spleen organ index in piglets, aligning with previous research on the utilization of herbal remedies to ameliorate the inflammatory response in weaned piglets (24).

The compromised immune system of early weaned piglets, owing to the ongoing development of their immune organs and weakened immune function, renders them susceptible to diseases (25). Maternal antibody immunity, encompassing the transmission of an animal’s immunoglobulins from the mother to the offspring via the placenta and breast milk, plays a pivotal role in bolstering the immune defence of piglets in early life (26). The cessation of immunoglobulin consumption following weaning increases the susceptibility of the piglet’s intestinal tract to infection by pathogenic microorganisms (27). As a rule, the IgG and IgA are frequently employed as markers to assess the impact of maternally acquired antibodies on the immune function of the offspring, whereas IgM serves as the primary defence against infections in the early stages of humoral immune response (28, 29). The immunomodulatory properties of plant extracts have been observed to activate immune function in weaned piglets (30). Our findings indicate that the administration of plant extract-containing PCE diets resulted in an increase in serum IgA, IgG, and IgM levels in weaned piglets. Furthermore, a dose-dependent effect was observed, suggesting that higher doses of PCE positively influenced the immune function of weaned piglets.

It is important to note that intestinal mucosal immune function plays a crucial role in protecting the intestine against pathogenic microorganisms (31). Past research has demonstrated that piglets, when exposed to immunological challenges and stress following weaning, experience the infiltration of endotoxins through the compromised intestinal barrier into the bloodstream. This infiltration triggers an elevation in the secretion of cytokines, including interleukin 1β (IL-1β) and interleukin 6 (IL-6), through the activation of the NF-κB signaling pathway (32). Notably, the administration of *Pulsatilla chinensis*, *Chinese Schneid*, and *Astragalus* extracts has been found to mitigate the inflammatory responses induced by endotoxins by inhibiting the reduction in cytokine secretion in various inflammatory models (33–35). Toll-like receptors (TLRs) serve as initiators of innate immunity by recognizing lipopolysaccharides, subsequently initiating a cascade of cellular signals involving MYD88 and ultimately activating the NF-κB signalling pathway (36). NF-κB, in turn, governs the inflammatory response by binding to nucleotide sequences located upstream of pro-inflammatory cytokine and chemokine genes (37). Our study revealed that PCE resulted in the downregulation of gene expression related to the colonic *TLR5* and MyD88/NF-κB pathways, ultimately leading to a decrease in levels of the proinflammatory factor *IL-6*. Hence, it is plausible that PCE exerts regulatory effects on the inflammatory response in weaned piglets via the TLR/MyD88/NF-κB pathway, as evidenced by the downregulation of pro-inflammatory cytokine gene expression level by PCE.

The establishment of a well-developed intestinal epithelium and a beneficial intestinal microbial community are crucial determinants in safeguarding animals against pathogenic bacterial infections (38). Intestinal villus height and crypt depth serve as indicators of intestinal nutrient absorption capacity and overall intestinal health, respectively (39). In contrast, low feed intake of piglets after early weaning adversely affects villus height and leads to frequent diarrhoea (40). The greater ease at which toxins enter the bloodstream as a result of morphological damage to the intestinal epithelium leads to the occurrence of a systemic inflammatory response (41). The inclusion of astragalus polysaccharide and berberine hydrochloride in the diet resulted in notable changes in the intestinal morphology of the animals, particularly in terms of villus height and crypt depth (42, 43). This observation implies that the active constituents present in these plants convey a beneficial impact on the gastrointestinal well-being and digestive health of piglets. The application of PCE in piglets resulted in an observed increase in ileal VH and a decrease in colonic CD, the ADFI of piglets from PCE groups was also enhanced. This suggests that PCE has the potential to enhance intestinal absorptive capacity and improve colonic health. It is worth noting that the increase in piglet feed intake might also indirectly reflect the strength. The intestinal flora, a crucial element of the intestinal barrier, exerts an immunosuppressive effect by secreting anti-inflammatory substances, with VFA being the main fermentation product (44). VFAs play a role in maintaining a dynamic equilibrium of the intestinal flora by reducing the pH, thereby enhancing the host’s resistance to pathogens and preventing adverse inflammatory responses (45). The combination of *Astragalus* polysaccharide and *Codonopsis* polysaccharide has been shown to re-establish immune homeostasis by increasing butyric, valeric, and isovaleric acid levels in the faeces of DSS-induced ulcerative colitis mice and restoring the composition of the intestinal flora (46). The supplementation of berberine hydrochloride has demonstrated the ability to enhance the content of VFAs as colonic metabolites through the modulation of the intestinal flora (47). Specifically, the administration of 500 mg/kg of a plant crude extract (PCE) resulted in elevated levels of valeric acid and isovaleric acid within the colonic content of weaned piglets. This increase in VFAs may be directly associated with the observed decrease in piglet diarrhoea rates and the subsequent enhancement of production performance.

Various saponins have demonstrated the ability to enhance microbial diversity (48). Additionally, *Astragalus* polysaccharide and berberine hydrochloride have been found to strengthen the intestinal barrier’s function by intervening to augment intestinal microbial diversity and facilitate the proliferation of advantageous bacteria (49). A robust gut microbial community co-evolves with the immune system, thereby impeding the infiltration of detrimental pathogens within the gut (50). Consequently, the enhancement of microbial diversity through the supplementation of dietary PCE proves advantageous for gut health. At the family level, piglets in the PCEH group demonstrated a heightened prevalence of the colonic microorganisms *Veillonellaceae* and *Rhodospirillales*. Similarly, at the genus level, the PCEH group exhibited a greater relative abundance of *Rhodospirillales* compared to the control group. Conversely, the relative abundance of *Eubacterium ventriosum* displayed an inverse pattern. *Veillonellaceae* primarily functions in lactic acid metabolism and enhances fat absorption by facilitating the dihydroxylation of bile acids (51). *Rhodospirillales*, a significant family of α-*Ascomycetes*, has been demonstrated to exert a beneficial influence on the modulation of the inflammatory response. Notably, the presence of *Rhodospirillales* was observed to be diminished in the intestinal microflora of afflicted mice in both a mouse model of colon cancer and a model of hypoxia-induced pulmonary hypertension (52, 53). *Eubacterium ventriosum*, a cluster of microorganisms closely linked to hindgut inflammation, exhibited a decrease in relative abundance in the gut of type 2 diabetic rats following treatment with extracts of *Astragalus* and *Rhizoma coptidis* (54). Furthermore, it was observed that the abundance of *Eubacterium ventriosum* exhibited a negative correlation with the levels of docosapentaenoic acid, a gut metabolite linked to inflammatory bowel disease (55). Consequently, the introduction of dietary PCE supplementation increases the diversity of the colonic microbiota and mitigates colonic inflammation and the immune response by facilitating the up-regulation of *Veillonellaceae* and *Rhodospirillales* at the family level, as well as inducing alterations in *Rhodospirillales* and *Eubacterium ventriosum* at the genus level.

## Conclusion

In summary, the findings of this study indicate that the inclusion of PCE in the diet resulted in a reduction in the incidence of diarrhoea and enhanced growth performance in weaned piglets. Furthermore, PCE exhibited efficacy as a supplement by enhancing the immune function of weaned piglets, potentially attributed to improvements in jejunal and colonic morphology, increased levels of intestinal VFA, and led to compositional alterations in colonic microorganisms. Notably, the dosage of 500 mg/kg demonstrated superior effects.

## Data availability statement

The datasets presented in this study can be found in online repositories. The names of the repository/repositories and accession number(s) can be found below: https://www.ncbi.nlm.nih.gov/, PRJNA1044009.

## Ethics statement

The animal study was approved by The Animal Ethics Committee of the Institute of Animal Sciences, Chinese Academy of Agricultural Sciences approved the experimental protocol (Ethics Approval Code: IAS2022-155). The study was conducted in accordance with the local legislation and institutional requirements.

## Author contributions

CX: Data curation, Writing – original draft. KL: Investigation, Writing – review & editing. CT: Investigation, Writing – review & editing. ZW: Project administration, Writing – review & editing. JL: Writing – review & editing. SZ: Writing – review & editing. LL: Writing – review & editing. HL: Project administration, Writing – review & editing. RZ: Project administration, Supervision, Writing – review & editing.
